# Novel Recombinant Newcastle Disease Virus-Based *In Ovo* Vaccines Bypass Maternal Immunity to Provide Full Protection from Early Virulent Challenge

**DOI:** 10.3390/vaccines9101189

**Published:** 2021-10-15

**Authors:** Kiril M. Dimitrov, Tonya L. Taylor, Valerie C. Marcano, Dawn Williams-Coplin, Timothy L. Olivier, Qingzhong Yu, Robert M. Gogal, David L. Suarez, Claudio L. Afonso

**Affiliations:** 1 Exotic and Emerging Avian Viral Disease Research Unit, Southeast Poultry Research Laboratory, US National Poultry Research Center, ARS, USDA, 934 College Station Road, Athens, GA 30605, USA or dimitrov_kiril@yahoo.com (K.M.D.); tonyaj@g.clemson.edu (T.L.T.); Marcano_Valerie@Elanco.com (V.C.M.); dawn.williamscoplin@usda.gov (D.W.-C.); tim.olivier@usda.gov (T.L.O.); david.suarez@usda.gov (D.L.S.); 2 Texas A&M Veterinary Medical Diagnostic Laboratory, 483 Agronomy Road, College Station, TX 77843, USA; 3 Endemic Poultry Viral Diseases Research Unit, Southeast Poultry Research Laboratory, US National Poultry Research Center, ARS, USDA, 934 College Station Road, Athens, GA 30605, USA; qingzhong.yu@usda.gov; 4 Department of Veterinary Biosciences & Diagnostic Imaging, College of Veterinary Medicine, The University of Georgia, Athens, GA 30602, USA; rgogal@uga.edu

**Keywords:** Newcastle disease, *in ovo* vaccine, maternal immunity, recombinant, cytokine, dose

## Abstract

Newcastle disease (ND) is one of the most economically important poultry diseases. Despite intensive efforts with current vaccination programs, this disease still occurs worldwide, causing significant mortality even in vaccinated flocks. This has been partially attributed to a gap in immunity during the post-hatch period due to the presence of maternal antibodies that negatively impact the replication of the commonly used live vaccines. *In ovo* vaccines have multiple advantages and present an opportunity to address this problem. Currently employed *in ovo* ND vaccines are recombinant herpesvirus of turkeys (HVT)-vectored vaccines expressing Newcastle disease virus (NDV) antigens. Although proven efficient, these vaccines have some limitations, such as delayed immunogenicity and the inability to administer a second HVT vaccine post-hatch. The use of live ND vaccines for *in ovo* vaccination is currently not applicable, as these are associated with high embryo mortality. In this study, recombinant NDV-vectored experimental vaccines containing an antisense sequence of avian interleukin 4 (IL4R) and their backbones were administered *in ovo* at different doses in 18-day-old commercial eggs possessing high maternal antibodies titers. The hatched birds were challenged with virulent NDV at 2 weeks-of-age. Post-hatch vaccine shedding, post-challenge survival, challenge virus shedding, and humoral immune responses were evaluated at multiple timepoints. Recombinant NDV (rNDV) vaccinated birds had significantly reduced post-hatch mortality compared with the wild-type LaSota vaccine. All rNDV vaccines were able to penetrate maternal immunity and induce a strong early humoral immune response. Further, the rNDV vaccines provided protection from clinical disease and significantly decreased virus shedding after early virulent NDV challenge at two weeks post-hatch. The post-challenge hemagglutination-inhibition antibody titers in the vaccinated groups remained comparable with the pre-challenge titers, suggesting the capacity of the studied vaccines to prevent efficient replication of the challenge virus. Post-hatch survival after vaccination with the rNDV-IL4R vaccines was dose-dependent, with an increase in survival as the dose decreased. This improved survival and the dose-dependency data suggest that novel attenuated *in ovo* rNDV-based vaccines that are able to penetrate maternal immunity to elicit a strong immune response as early as 14 days post-hatch, resulting in high or full protection from virulent challenge, show promise as a contributor to the control of Newcastle disease.

## 1. Introduction

Newcastle disease (ND), a devastating poultry disease that can reach 100% mortality in naïve birds, is caused by virulent strains of Newcastle disease virus (NDV) [1]. This virus species, recently renamed *Avian orthoavulavirus 1*, is a member of the family *Paramyxoviridae* [2,3]. Newcastle disease viruses have a single stranded, non-segmented, negative sense RNA genome encoding for at least six structural proteins (3′—nucleoprotein [NP]—phosphoprotein [P]—matrix protein [M]—fusion protein [F]—hemagglutinin-neuraminidase [HN]—and large RNA-polymerase [L]—5′) and comprising one of three genome sizes (15,186, 15,192, and 15,198 nucleotides) [4,5]. Newcastle disease is considered the third most important poultry disease worldwide [6] and remains endemic in many countries throughout Asia, Africa, and the Americas [7].

The control of ND includes strict biosecurity to prevent the introduction of virulent NDV (vNDV) onto poultry farms [8,9] and proper administration of vaccines [10]. However, the multiple ND outbreaks occurring worldwide [11] are suggestive that the current vaccines and vaccination procedures are not fully efficacious. In addition to the conventional live and inactivated vaccines, multiple concepts for ND vaccination have been explored in the last two decades, including vectored, antigen–antibody complex, virus-like particle, and toll-like receptor ligand vaccines, among others [1,8,9]. Several avian vaccines, engineered to co-express immunostimulatory cytokines, have also been suggested to improve protective immunity [12]. Another approach that has been explored is the use of antigenically matched vaccines. Although all NDV are regarded as members of a single serotype [3,13], there is antigenic variation between strains [14]. Previous studies with chickens of different ages have demonstrated that chickens vaccinated with strains that were antigenically matched to the challenge virus shed significantly less amount of the challenge vNDV than the amount excreted by chickens vaccinated with heterologous vaccines [15,16,17]. Of note, passively transferred maternal anti-NDV antibodies, although protecting chickens during the crucial early weeks of life, interfere with development of host immunity following vaccination [18].

*In ovo* vaccination is an approach that delivers multiple advantages—for example, significant cost reduction, standardization, mass vaccine application, automated vaccination at the hatchery, uniform delivery of the vaccine in each egg, and no stress to birds [19,20,21]—and is an attractive immunization method for the poultry industry [22]. Between 80% and 90% of the broiler chickens grown in the U.S. are currently *in ovo*-vaccinated two to three days prior to hatch [23,24], and the automation of the process allows for up to 70,000 eggs to be vaccinated per hour [23]. Globally, this vaccine administration method is employed in 26 out of the 30 leading poultry production countries in the world [23]. A few *in ovo* vaccines against ND are currently available, but they are all generated using non-NDV vectors. Fowlpox virus (FPV) vector-based vaccines expressing the NDV F and HN proteins applied *in ovo* have been shown to protect poultry from a challenge with vNDV [25,26]. However, recombinant FPV-ND vaccines are not widely used due to immune interference from previous FPV vaccinations or exposures (FPV is commonly present in the environment) and lack of optimization for application through mass methods [9]. The most widely used vector for recombinant ND *in ovo* vaccine production is the Meleagrid alphaherpesvirus 1, commonly known as herpesvirus of turkeys (HVT) or a serotype 3 Marek’s disease virus [12,23]. In the U.S. alone, 8.2 billion doses of recombinant HVT (rHVT)-ND vaccines were produced in 2018 compared with 3.6 billion doses of conventional ND vaccines [27]. In addition to the advantage of being used *in ovo*, the rHVT vaccines expressing NDV proteins elicit strong and long-term immunity following a single application [28]. Further, they are transiently affected by the presence of anti-NDV maternally derived antibodies [8]. However, the rHVT-ND vaccines have some limitations, such as delayed immunity, and a previous HVT vaccination appears to be refractory to the use of booster HVT vaccines in the same birds [9,29]. The rHVT vaccines are cell-associated and are required to be stored and transported in liquid nitrogen, which is a limitation on their use in many countries.

The prospect for new *in ovo* vaccines is growing [23]. Different vectors have been explored for the *in ovo* route of administration, and promising results have been reported with non-replicating human adenovirus- [30] and alphavirus-vectored vaccines [31]. Although there have been no advancements to routine commercial use, efforts have also been made for development of novel *in ovo* non-HVT NDV-vectored vaccines [32,33,34,35]. Employing live ND vaccines *in ovo* is a challenge. While live ND vaccines, such as LaSota, B1, Clone 30, and Ulster, to name a few, confer a strong immune response and are commonly used for vaccination of chickens as young as one day old, they are associated with high embryo mortality when administered at 18 days of embryonation. Mortality as high as 100% using wild-type low virulent viruses and 80% using genetically attenuated live viruses have been reported after *in ovo* use, and no live ND vaccines are currently used for this method of vaccine administration [36,37].

A novel recombinant NDV (rNDV)-vectored experimental vaccine containing an antisense avian interleukin 4 insert (IL4), named ZJ1*l-IL4R, was recently shown to be a promising *in ovo* vaccine candidate after evaluation in specific-pathogen-free (SPF) embryonating chicken eggs (ECE) [38]. The aim of this study was to further evaluate the applicability of this recombinant NDV-vectored vaccine and its recombinant backbone (attenuated cleavage site from wild-type virus) and a recombinant LaSota-IL4R virus for *in ovo* vaccination of 18-day-old commercial eggs with high maternal antibody titers. Commercially available vaccines LaSota and rHVT-ND were included as controls. In addition, this study aimed to evaluate the protection efficacy of the studied vaccines against early challenge with vNDV. Here, the ability of these recombinant vaccines to efficiently overcome maternal immunity, even in low doses, and to induce a strong immune response is demonstrated. The experimental rNDV vaccines demonstrated 100% protection after early challenge with vNDV.

## 2. Materials and Methods

### 2.1. Viruses

The virulent goose/China/ZJ1/2000 (ZJ1, GenBank accession number AF431744) NDV, member of sub-genotype VII.1.1 (former VIId), was used as a challenge virus in this study. A recombinant ZJ1 virus (attenuated version of ZJ1) with a low virulent cleavage site (ZJ1*L) that was previously generated in our laboratory through reverse genetics [39] was used as a backbone for creating recombinant ZJ1*L vaccines and as a vaccine virus. The low virulent LaSota (LS) vaccine strain was used as a vaccine control and as a backbone to generate a recombinant LS-IL4R vaccine. All viruses were obtained from the Southeast Poultry Research Laboratory’s (SEPRL) repository of the United States National Poultry Research Center (USNPRC), U. S. Department of Agriculture (USDA). Additionally, a commercially available rHVT-ND vaccine (fusion gene insert) was also used as a control.

The recombinant NDV experimental vaccines used in this study were created using reverse genetics, as previously described [38,39]. Briefly, the coding sequence of avian IL4 was cloned into the backbones of ZJ1*L and LS in the reverse orientation (IL4R) between the P and M genes, generating two recombinant attenuated live viruses—namely, ZJ1*L-IL4R and LS-IL4R (GenBank accession numbers MM680659 and MM680665, respectively). The recombinant viruses were rescued using the modified vaccinia virus Ankara expressing the T7 polymerase (MVA/T7), following previously published protocols [38,39,40,41]. A schematic representation of the constructs is provided in Supplementary Figure S1A. For the purpose if this work, the term “rNDV vaccines” used hereafter refers to ZJ1*L, ZJ1*L-IL4R, and LS-IL4R.

Working stocks of all NDVs were propagated in 9- to 11-day-old SPF embryonating chicken eggs (ECE) [42]. Viral stocks used to inoculate eggs in this study were diluted in brain heart infusion (BHI) broth (BD Biosciences, MD, USA), containing 200 µg/mL gentamicin, 2000 U/mL penicillin, and 4 µg/mL amphotericin B, to three different target titers, 10^5.5^/0.1 mL, 10^4.5^/0.1 mL, and 10^3.5^/0.1 mL embryo infectious dose (EID_50_), except LS, which was diluted to 10^5.5^/0.1 mL and 10^4.5^/0.1 mL EID_50_. The titers of the diluted working stocks of the live vaccines were confirmed by back titrations in 9- to 11-day-old SPF ECEs following standard procedures, as referenced above. The rHVT-ND vaccine was directly diluted using the sucrose phosphate glutamate albumin diluent for rHVT vaccines described in the USDA’s Center for Veterinary Biologics Test Protocol [43].

### 2.2. Eggs

Two hundred and sixty-two commercial (non-SPF) eggs from the Hy-Line Rockside W-36 breed layer flock (Mansfield, GA, USA) were kindly donated for this experiment. The layer flock had been routinely vaccinated with the LaSota vaccine. The flock immunity has been regularly tested by ELISA, and although these results are not directly translatable to hemagglutination-inhibition (HI) titers, the average anti-NDV antibody levels of the parental flock were very high (i.e., 15,534 with a cutoff for positive samples of 1159). The source of SPF ECEs (42 eggs) was the SEPRL SPF White Leghorn flock. All eggs were incubated at 37.5 °C temperature and 55–60% relative humidity for 18 days.

### 2.3. Vaccination

The commercial (*n* = 262) and SPF (*n* = 42) eggs, at 18 days of embryonation (DOE), were separated into 14 groups with 21 eggs in each group (except BHI and Hatch control groups, which had 26 eggs each). Using 1 mL, 24 G × 1/2 syringes, each egg was inoculated into the allantoic cavity with 0.1 mL of diluted vaccine or BHI, except for the Hatch control group, in which the eggs were not manipulated at all. There were thirteen groups of inoculated eggs, separated into seven experimental and six control groups. The seven experimental groups were as follows: ZJ1*L-IL4R 10^4.5^, ZJ1*L-IL4R 10^5.5^, LS-IL4R 10^3.5^, LS-IL4R 10^4.5^, LS-IL4R 10^5.5^, ZJ1*L 10^4.5^, and ZJ1*L 10^5.5^, each receiving the respective EID_50_ dose of inoculum per 0.1 mL as stated in the group name. Additionally, six control groups were created. Two controls groups of commercial eggs were inoculated as described above with BHI and rHVT-ND, respectively. Two control groups of commercial eggs were inoculated as described above with LS 10^4.5^ and LS 10^5.5^ EID_50_ dose of inoculum per 0.1 mL. Two control groups of SPF eggs were inoculated with ZJ1*L 10^3.5^ and ZJ1*L-IL4R 10^3.5^. A schematic representation of the study design is provided in Supplementary Figure S1B,C.

After inoculation, the eggs were placed inside Turbofan Hova-Bator Incubators (GQF Manufacturing Company Inc., GA, USA) that were subsequently placed in isolators in an animal biosecurity level 2 (ABSL-2) facility. The incubators were regulated with thermostats to maintain a constant temperature of 37.5 °C and relative humidity between 55% and 60%.

### 2.4. Hatching, Sampling, Mortality

After hatch at 21 DOE, chicks were housed in negative pressure isolators (ABSL-2) and provided food and water with *ad libitum* access. Five non-SPF chickens from both the Hatch control and BHI groups were sacrificed and bled immediately post-hatch to establish the baseline titer of maternal anti-NDV HI antibodies. All chickens were weighed at 1, 8, and 13 days post-hatch (DPH). Oropharyngeal and cloacal swabs were collected in BHI from each bird at 2 and 4 DPH. Clinical signs and mortality were recorded twice daily throughout the post-hatch period, and blood samples were collected at 13 DPH.

### 2.5. Challenge

Twelve birds from each group (except LS 10^5.5^, in which all birds died by 6 DPH) were transferred to ABSL-3E facility at 13 DPH. The birds were placed into negative pressure isolators and allowed to acclimate for 24 h before challenge. All birds were challenged at 14 DPH with virulent ZJ1 NDV at 10^6^ EID_50_/0.1 mL by the oculo-nasal route. Birds were observed daily for clinical signs and mortality. Oropharyngeal and cloacal swabs were collected from each bird at 2 and 4 days post-challenge (DPC). All surviving birds were weighed and bled at 14 DPC and were euthanized by cervical dislocation after anesthesia with 0.2 mL ketamine/xylazine solution (66 mg/mL ketamine and 6.6 mg/mL xylazine) administered intramuscularly with 25-gauge needle.

### 2.6. Real-Time RT-PCR

RNA was extracted from all swab medium using the MagMAX 96 AI/ND viral RNA isolation kit (Ambion, Inc. Austin, TX, USA) with a KingFisher magnetic particle processor (Thermo Fisher Scientific, Waltham, MA, USA) following the manufacturer’s instructions, with the inhibitor removal modification described by Das et al. [44]. Quantitative real-time RT-PCR (rRT-PCR) for NDV detection targeting the matrix (M) gene was performed using AgPath-ID one-step RT-PCR Kit (Ambion) and the ABI 7500 Fast Real-Time PCR system (Applied Byosystems, Waltham, MA, USA) utilizing primers and probe previously described by Wise et al. [45]. The assay conditions were reverse transcription for 10 min at 45 °C; initial denaturation for 10 min at 95 °C; 40 cycles of denaturation for 10 sec at 94 °C, primer annealing for 30 sec at 56 °C, and primer elongation for 10 sec at 72 °C. The total reaction volume of 25 μL for each sample consisted of molecular grade water—2.5 μL; 2X buffer—12.5 μL; forward primer—0.25 μL (20pmol/μL); reverse primer—0.25 μL (20 pmol/μL); probe—0.50 μL (6pmol/μL); 25X enzyme mix—1.0 μL; and RNA template—8.0 μL. For virus quantification, a standard curve for each virus was established, with RNA extracted from the same titrated stock of the viruses used to inoculate the eggs (for the swab samples collected post-hatch) or the challenge virus (for the swab samples collected post-challenge). The results are reported as EID_50_/mL. The calculated lower detection limit of the assays was between 10^1.7^ and 10^1.9^ EID_50_/mL. It is important to note that rRT-PCR detects viral nucleic acids and that the estimated values do not necessarily represent infectious virus. The EID_50_/mL values do not aim to present infectiousness and are used to provide means for relative comparison of viral shedding between groups. Despite the use of inhibitor removal nucleic acids extraction protocol, the high volume of RNA template may have impacted the calculations from the standard curve. Of note, the same amount of RNA template was used in all reactions, and potential residue inhibitors would have impacted all results equally.

### 2.7. HI Assay

Hemagglutination inhibition (HI) assay was used to evaluate the presence and titers of antibodies in the serum samples collected throughout the experiment. Following standard procedures [46], the virus used as the backbone for each vaccine was used as an antigen for each respective group of sera (ZJ1*L for all ZJ1 groups and LS for all LS groups). For the serum samples collected post-challenge, the virulent ZJ1 virus was used as an antigen in the HI assay. Titers were calculated as the reciprocal of the last HI-positive serum dilution, and samples with HI titers of log_2_ 3 and below were considered negative.

### 2.8. Statistical Analyses

Prism v.7.03 (GraphPad Software Inc., La Jolla, CA, USA) software was used to analyze the data, and outliers were identified using the ROUT test. The D’Agostino–Pearson normality test was performed to estimate whether the values in each group came from a Gaussian distribution. Based on the normality distribution, non-parametric Kruskal–Wallis test with Dunn’s post-test was used for multiple comparisons between groups. For statistical purposes, all swab samples from which viruses were not detected were given a numeric value of 1 log below the limit of detection for each virus. The survival curves were analyzed using the Log-rank (Mantel–Cox) test. Statistical significance was set at a *p* value of < 0.05.

## 3. Results

### 3.1. Hatchability, Post-Hatch Survival, and Body Weights

**Figure 1:**
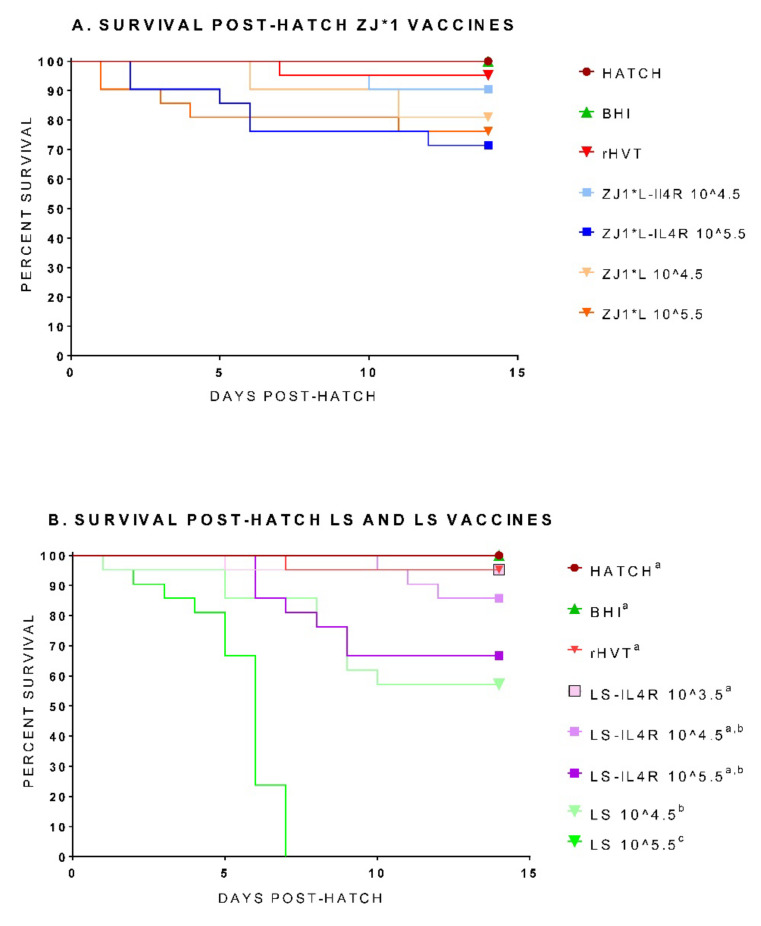
Survival of chickens post-hatch after inoculation of commercial eggs at 18 days of embryonation with experimental *in ovo* NDV vaccines. Hy-Line Rockside W-36 eggs were inoculated with recombinant viruses and LaSota. Three control groups were inoculated with BHI or rHVT-ND vaccine, or not inoculated (Hatch control group). Birds were monitored for 13 days post-hatch. The survival curves are presented in two figures: (**A**) control groups and ZJ1*L groups and (**B**) control groups and LS-IL4R groups. Different superscript letters indicate statistically significant differences between groups (*p* value < 0.05). Group abbreviations: HATCH—eggs not inoculated; BHI—eggs inoculated with BHI; rHVT-ND—eggs inoculated with recombinant HVT vaccine; ZJ1*L—eggs inoculated with the recombinant ZJ1*L virus whose cleavage site was modified to be of low virulence; ZJ1*L-IL4R—eggs inoculated with the recombinant low virulent ZJ1*L with the interleukin-4 gene inserted in reverse orientation; LS—eggs inoculated with the LaSota strain; LS-IL4R—eggs inoculated with the recombinant LaSota virus with the interleukin-4 gene inserted in reverse orientation. The virus dose that the eggs in each group received is represented as a number after the group name in EID_50_/per egg.

The post-hatch survival for all groups hatched from commercial eggs is depicted as survival curves in Figure 1 (separated in Figure 1A,B for clarity and to avoid congestion of the graphics). A combined graph is available in Supplementary Figure S2. The Hatch control group (not inoculated) was included in the design to ensure that there was no abnormal hatchability or post-hatch mortality that was not related to the inoculation of the eggs. As expected, very low (rHVT-ND) to no (Hatch and BHI) post-hatch mortality was observed in three of the control groups. In contrast, the LS control groups had 57.1% and 0% survival when the 10^4.5^ and 10^5.5^ doses were used, respectively, which has previously been reported for conventional ND live vaccines. Two eggs did not hatch in the LS-IL4R 10^5.5^ group and one egg did not hatch in the LS 10^4.5^ group. Variable mortality was observed in the other groups depending on the vaccine and inoculation dose. The ZJ1*L-IL4R 10^4.5^ and 10^5.5^ groups had 90.5% and 71.4% survival, respectively, and the survival recorded in the ZJ1*L 10^4.5^ and 10^5.5^ groups was 80.9% and 76.2%, respectively (Figure 1A), but the differences were not statistically significant. The survival in the LS-IL4R 10^3.5^ group was 95.2%. The survival rates observed in the LS-IL4R 10^4.5^ and 10^5.5^ groups were 85.7% and 66.7%, respectively (Figure 1B). There were no significant differences in survival between the experimental ZJ1*L, ZJ1*L-IL4R, and LS-IL4R vaccine groups and the Hatch, BHI, and rHVT-ND control groups, but the percentage survival in these control groups was numerically higher, with a negative correlation between vaccine dose and post-hatch survival observed (survival increased as vaccine dose decreased). The LS 10^5.5^ control group had significant differences in survival compared with the experimental groups (*p* values between 0.0001 and 0.02). The LS 10^4.5^ control group, although not statistically different, had substantially lower survival rates compared with the experimental groups with the same dose. The two LS control groups had significant differences in survival compared with the Hatch, BHI, and rHVT-ND control groups (*p* values between 0.0001 and 0.04). Clinical signs were not observed in any of the groups post-hatch. In the groups in which mortality post-hatch was present, the birds were found dead upon daily checks.

All birds were weighed at days 1, 8, and 13 post-hatch (Table 1). At 1 DPH, the average body weight per bird in most groups was between 38.5 and 42.6 grams (g). There were significant body weight differences only between rHVT-ND and ZJ1*L-IL4R 10^4.5^ groups from one side and ZJ1*L 10^4.5^ and control LS 10^4.5^ from another (the birds in the two first groups weighed more than the birds in the control group) (*p* values between 0.0001 and 0.017, respectively). At 8 DPH, the two ZJ1*L groups and the LS-IL4R 10^4.5^, the LS-IL4R 10^5.5^ group, and the control LS group (average weight 50–63 g) had body weights significantly lower compared with the birds from the Hatch, BHI, and rHVT-ND control groups (average weight 77–82 g) (*p* values between 0.0001 and 0.03). The same groups that were significantly different from the controls at 8 DPH (except LS-IL4R 10^4.5^) had significantly lower body weights than the control Hatch, BHI, and rHVT-ND groups at 13 DPH too (*p* values between 0.0001 and 0.01). The birds from the inoculated SPF control groups were not included in this comparison, as SPF eggs (and the birds hatched from them) are traditionally smaller and lighter than commercial eggs.

### 3.2. Shedding of Vaccine Viruses Post-Vaccination, as Demonstrated by rRT-PCR

**Figure 2:**
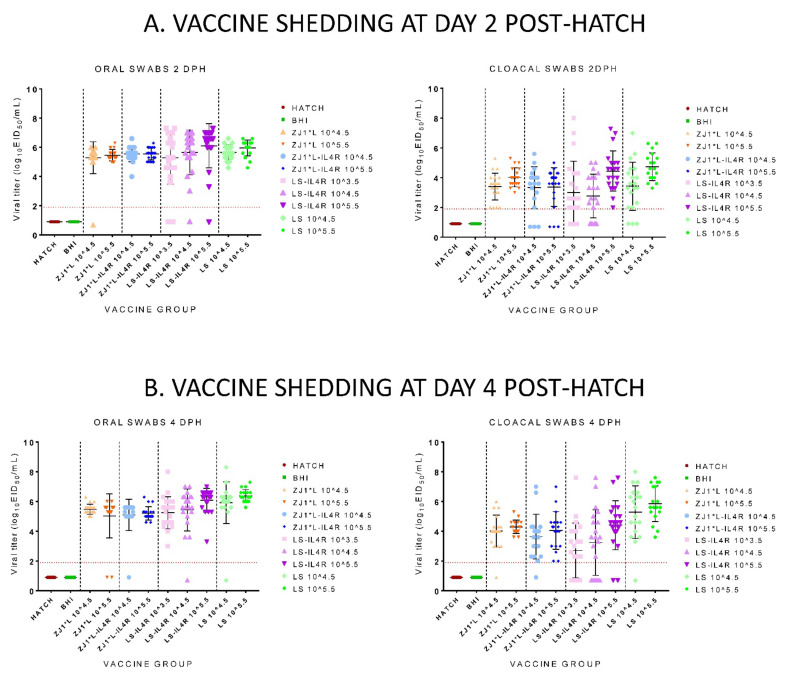
Mean post-hatch vaccine shedding from chickens hatched from commercial eggs inoculated at 18 days of embryonation with experimental *in ovo* vaccines. Each data point represents one bird and NDV titers detected in oropharyngeal (OP) and cloacal (CL) swabs at different days post-hatch (DPH). Shedding is presented separately for 2 days post-hatch (**A**) and 4 days post-hatch (**B**). Bars represent standard deviations of the mean. All swabs from which virus was not detected were given a numeric value of 1 log below the limit of detection for each of the respective viruses. The limit of detection of rRT-PCR is presented as a horizontal dotted line (the lowest limit is used). Group abbreviations: HATCH—eggs not inoculated; BHI—eggs inoculated with BHI; rHVT-ND—eggs inoculated with recombinant HVT vaccine; ZJ1*L—eggs inoculated with the recombinant ZJ1*L virus whose cleavage site was modified to be of low virulence; ZJ1*L-IL4R—eggs inoculated with the recombinant low virulent ZJ1*L with the interleukin-4 gene inserted in reverse orientation; LS—eggs inoculated with the LaSota strain; LS-IL4R—eggs inoculated with the recombinant LaSota virus with the interleukin-4 gene inserted in reverse orientation. The virus dose that the eggs in each group received is represented as a number after the group name in EID_50_/per egg.

At 2 DPH, there were no significant differences in viral shedding among vaccine groups. (Figure 2A). Similarly, at 4 DPH, the differences between groups were not statistically significant. (Figure 2B). No viral shedding was detected in any of the Hatch and BHI control birds.

### 3.3. Humoral Immune Response Post-Hatch

**Figure 3:**
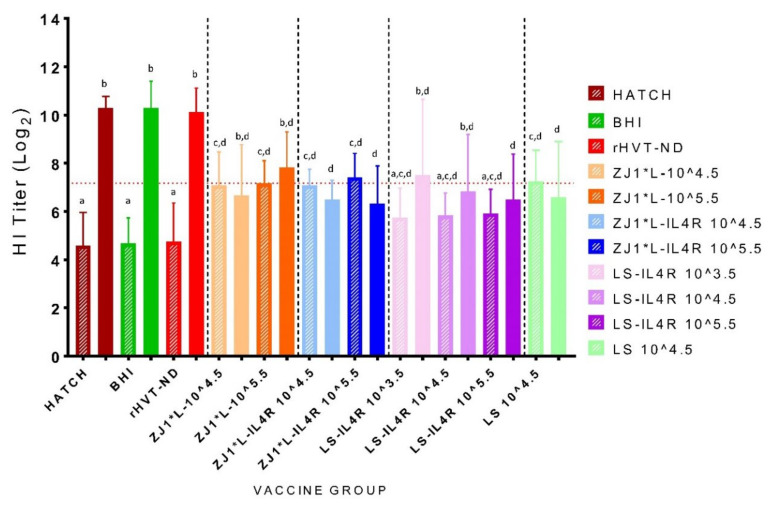
Pre- and post-challenge HI antibody titers (and standard deviation) from chickens hatched from commercial eggs inoculated at 18 days of embryonation with experimental *in ovo* vaccines and early challenge with virulent Newcastle disease virus at 14 days post-hatch.The diagonal stripe-filled bars represent the pre-challenge titers at 13 days post-hatch. The solid-filled bars represent the post-challenge titers at 14 days post-challenge. Statistically significant differences between the different groups are presented with lowercase letters above each group (*p* value <0.05). The level of maternal antibodies at the day of hatch (0 DPH) is presented as a horizontal dotted line. Group abbreviations: HATCH—eggs not inoculated; BHI—eggs inoculated with BHI; rHVT-ND—eggs inoculated with recombinant HVT vaccine; ZJ1*L—eggs inoculated with the recombinant ZJ1*L virus whose cleavage site was modified to be of low virulence; ZJ1*L-IL4R—eggs inoculated with the recombinant low virulent ZJ1*L with the interleukin-4 gene inserted in reverse orientation; LS—eggs inoculated with the LaSota strain; LS-IL4R—eggs inoculated with the recombinant LaSota virus with the interleukin-4 gene inserted in reverse orientation. The virus dose that the eggs in each group received is represented as a number after the group name in EID_50_/per egg.

Analysis of serum samples collected at 13 DPH (diagonal stripe-filled bars) by HI assay (detecting hemagglutination antibodies to NDV) is depicted in Figure 3. To establish the titer of passively transferred anti-NDV maternal HI antibodies in the commercial eggs, ten chickens were bled immediately after hatch (0 DPH). The HI titers at hatch varied between log_2_ 5 and log_2_ 8, with an average of log_2_ 7.17 (Figure 3, dotted horizontal red line). The antibodies at 13 DPH in the Hatch, BHI, and rHVT-ND groups (average of log_2_ 4.7) are residual maternal antibodies, as these groups were either not vaccinated or vaccinated with rHVT-ND vaccine, which does not induce HI antibodies to NDV. The antibody titers in the ZJ1*L, ZJ1*L-IL4R, and the LS 10^4.5^ groups were significantly higher compared with the Hatch, BHI, and rHVT-ND control groups. No significant differences between the different dose groups of the vaccines with the same backbone were observed. The LS-IL4R groups had lower post-vaccination HI titers compared with the ZJ1*L-IL4R and ZJ1*L groups, although these differences were not statistically significant. Of note, although numerically slightly higher, the post-vaccination HI titers of the birds in all LS-IL4R groups were not significantly different from the titers measured in the control unvaccinated birds.

### 3.4. Clinical Signs and Survival after Early Challenge with vNDV

In the control groups, the first clinical signs to be observed were in the Hatch and BHI groups at 3 DPC. Three birds in the Hatch group and one bird in the BHI group had labored breathing. At 4 DPC, ruffled feathers and head tremors were seen in the BHI and hatch groups and at 5 DPC in the rHVT-ND group; in the latter group, ataxia and severe torticollis were also recorded in two birds. The birds in the Hatch control group presented lethargy at 5 DPC, and two birds in the BHI group showed paralysis. Lethargy and paralyses were observed in the rHVT-ND group at 6 and 7 DPC. Opisthotonos, torticollis, and labored breathing were observed in the Hatch and BHI control groups beginning at 9 DPC. In the rHVT-ND group, torticollis was observed as early as 5 DPC, labored breathing and ataxia began at 6 DPC, and opisthotonos began at 13 DPC. By 14 DPC, all birds except two Hatch, one BHI, and two rHVT-ND birds (83.3%, 91.6%, and 83.3% morbidity, respectively) either died or developed predominantly neurological clinical signs such as ataxia, opisthotonus, torticollis, tremors, and paralyses. Birds that showed severe clinical signs, stopped eating or drinking, or remained recumbent were euthanized and reported as dead on the next day for the calculation of survival times. No clinical signs were observed in any of the ZJ1*L and ZJ1*L-IL4R groups. Slight clinical signs of opisthotonos or torticollis were observed in a single bird (8.4% morbidity) in each of the LS-IL4R groups and the LS 10^4.5^ group at 14 DPC.

**Figure 4:**
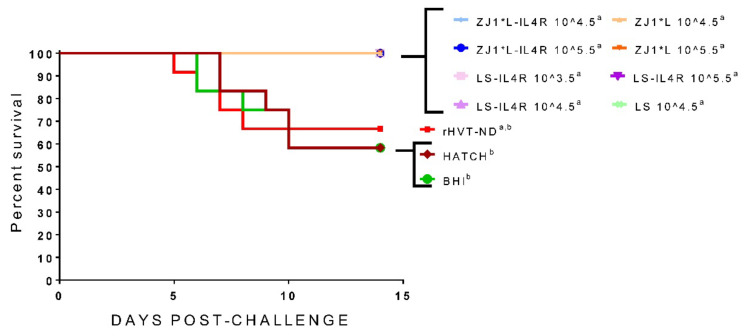
Survival of chickens after inoculation of commercial eggs at 18 days of embryonation with experimental *in ovo* vaccines and early challenge with virulent Newcastle disease virus at 14 days post-hatch. Birds were monitored for 14 days post-challenge. Different superscript letters indicate statistically significant differences between groups (*p* value < 0.05). Group abbreviations: HATCH—eggs not inoculated; BHI—eggs inoculated with BHI; rHVT-ND—eggs inoculated with recombinant HVT vaccine; ZJ1*L—eggs inoculated with the recombinant ZJ1*L virus whose cleavage site was modified to be of low virulence; ZJ1*L-IL4R—eggs inoculated with the recombinant low virulent ZJ1*L with the interleukin-4 gene inserted in reverse orientation; LS—eggs inoculated with the LaSota strain; LS-IL4R—eggs inoculated with the recombinant LaSota virus with the interleukin-4 gene inserted in reverse orientation. The virus dose that the eggs in each group received is represented as a number after the group name in EID_50_/per egg.

Survival post-challenge with the virulent ZJ1 NDV is depicted as survival curves in Figure 4. While all groups inoculated with the recombinant vaccines and LS showed 100% survival, the birds in the Hatch, BHI, and rHVT-ND groups had 58.3%, 58.3%, and 66.6% survival, respectively. The post-challenge survival in the Hatch and the BHI control groups was significantly lower compared with the experimental vaccine groups. Of note, although the body weights of the birds in the inoculated groups fluctuated post-hatch compared with the Hatch, BHI, and rHVT-ND controls, all rNDV vaccinated birds (282–328 g) surpassed the body weights of the non-vaccinated control birds post-challenge (255–266 g) (Table 1). However, no significant body weight difference was observed between any of the studied groups.

### 3.5. Shedding Post-Challenge as Demonstrated by rRT-PCR

**Figure 5:**
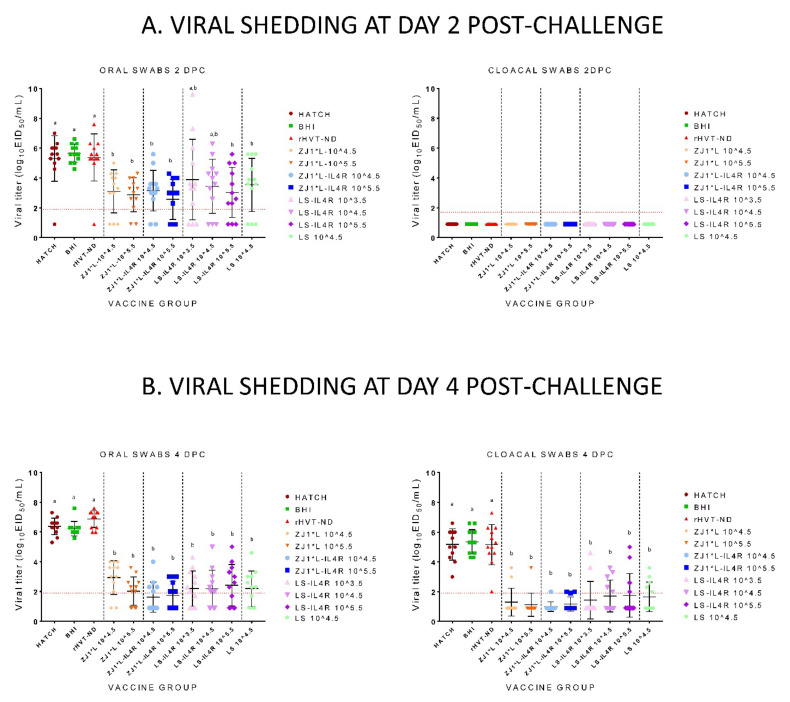
Mean post-challenge viral shedding from chickens after inoculation of commercial eggs at 18 days of embryonation with experimental *in ovo* vaccines and early challenge with virulent Newcastle disease virus at 14 days post-hatch. Each data point represents NDV titers detected in oropharyngeal (OP) and cloacal (CL) swabs at different days post-challenge (DPC). Shedding is presented separately for 2 days post-challenge (**A**) and 4 days post-challenge (**B**). Bars represent standard deviations of the mean. All swabs from which virus was not detected were given a numeric value of 1 log below the limit of detection. The limit of detection of rRT-PCR is presented as a horizontal dotted line. Statistically significant differences between the different groups are presented with lowercase letters above each group (*p* value < 0.05). Group abbreviations: HATCH—eggs not inoculated; BHI—eggs inoculated with BHI; rHVT-ND—eggs inoculated with recombinant HVT vaccine; ZJ1*L—eggs inoculated with the recombinant ZJ1*L virus whose cleavage site was modified to be of low virulence; ZJ1*L-IL4R—eggs inoculated with the recombinant low virulent ZJ1*L with the interleukin-4 gene inserted in reverse orientation; LS—eggs inoculated with the LaSota strain; LS-IL4R—eggs inoculated with the recombinant LaSota virus with the interleukin-4 gene inserted in reverse orientation. The virus dose that the eggs in each group received is represented as a number after the group name in EID_50_/per egg.

At 2 DPC, the birds in the Hatch, BHI, and rHVT-ND control groups shed high viral titers (10^5.3−5.7^ EID_50_/mL) through the oral route, while all vaccine groups had lower than 10^4^ EID_50_/mL mean shedding titers (Figure 5A). The results for viral shedding through the cloaca were below the detection limit of the assay in all groups. At 2 DPC, the birds in all ZJ1*L and ZJ1*L-IL4R groups and those in the LS-IL4R 10^5.5^ group had significantly less viral RNA detected though the oral route compared with the Hatch, BHI, and rHVT-ND control groups (*p* values ranged between 0.001 and 0.02). At 4 DPC, the shedding through the oral route in the Hatch, BHI, and rHVT-ND groups was high (10^6.2−6.9^ EID_50_/mL) (Figure 5B). In contrast, less shedding was observed across all vaccine groups at 4 DPC compared with 2 DPC, with the highest mean titer of 10^2.9^ EID_50_/mL in the ZJ1*L 10^4.5^ group and the lowest mean titer of 10^1.5^ EID_50_/mL in the ZJ1*l-IL4R 10^4.5^ group (viral RNA was not detected in some birds). All experimental rNDV vaccine groups shed significantly less virus through the oral route compared with the Hatch, BHI, and rHVT-ND control groups (*p* values from <0.0001 to 0.03). No significant differences in oral shedding were observed among the experimental rNDV vaccine groups. The birds in the rNDV vaccine groups shed significantly less virus through the cloacal route compared with the Hatch, BHI, and rHVT-ND control groups (*p* values from <0.0001 to 0.007). The differences in the titers were not significant among the experimental rNDV vaccine groups and the LS control group.

### 3.6. Post-Challenge Serology

All birds in the Hatch, BHI, and rHVT-ND control groups showed increased (more than double) HI-antibody titers at 14 DPC (above log_2_ 10) compared with the pre-challenge titers at 13 DPH (log_2_ 4.7). The differences of the titers in these three groups pre- and post-challenge were significant (*p* value <0.0001) (Figure 3, solid bars). In contrast, the post-challenge HI titers in all experimental rNDV vaccine groups and the LS control group did not change significantly compared with the pre-challenge titers of the same groups. In all groups, the differences between pre- and post-challenge antibody titers were within a log (except LS-ILR4 10^3.5^, in which the difference was log_2_ 1.8).

### 3.7. Inoculated SPF Control Groups

The survival in the SPF control groups post-hatch was 66.7% in the group inoculated with ZJ1*L 10^3.5^ and 57.1% in the group inoculated with ZJ1*L-IL4R 10^3.5^ (Supplementary Figure S3A). Active viral shedding was detected in the birds in the SPF control groups through both routes, with higher titers detected in the oropharyngeal swab samples (see Supplementary Figure S3B). The post-vaccination HI titers of the inoculated SPF control birds were higher than the titers in the Hatch, BHI, and rHVT-ND control groups and reached mean values of log_2_ 7.08 and log_2_ 7.58 at 13 DPH in the ZJ1*L-IL4R 10^3.5^ and ZJ1*L-IL4R 10^3.5^ groups, respectively (see Supplementary Figure S3C). No post-challenge clinical signs or mortality were observed in the two inoculated SPF control groups (see Supplementary Figure S3D for survival curves). The birds from the inoculated SPF control groups shed significantly less virus through the oral route at 2 DPC and 4 DPC compared with the control groups (*p* values from <0.0001 to 0.009) (see Supplementary Figure S3E). The post-challenge antibody titers in both inoculated SPF control groups did not change significantly compared with the pre-challenge titers of the same groups (see Supplementary Figure S3C).

## 4. Discussion

Despite the wide use of vaccination programs in commercial flocks, virulent Newcastle disease viruses continue to cause disease outbreaks globally [11]. The frequent occurrence of outbreaks and lack of complete efficacy of the currently available vaccines indicate that development of alternative approaches for vaccine generation is probably needed. Here, the immunogenicity of two recombinant NDV-vectored experimental vaccines, containing an antisense avian IL4 insert and their backbone viruses for *in ovo* vaccination of 18-day-old commercial eggs with high maternal immunity, and their protection efficacy against challenge with vNDV were evaluated. These recombinant vaccines efficiently overcame maternal immunity and elicited a strong post-vaccination immune response. The experimental vaccines provided 100% protection after early challenge with vNDV. Of note, the live LaSota low virulent virus, widely used as conventional ND vaccine in chickens, induced humoral immune response and provided protection from clinical disease after early vNDV challenge in the *in ovo*-vaccinated birds that survived post-hatch. However, the post-hatch mortality after *in ovo* vaccination with LaSota was very high—100% in the LS-10^5.5^ group and 43% in the LS-10^4.5^ group. In addition, there were significant differences in body weights compared with the non-vaccinated control groups and, although low, 8.4% morbidity after challenge. The high mortality and differences in body weights render this vaccine not applicable for *in ovo* vaccination, as these adverse effects will result in significant economic losses when scaled to industrial poultry settings.

The recombinant ZJ1*L and ZJ1*L-IL4R vaccines administered *in ovo* at 18 DOE efficiently induced a strong humoral immune response in the presence of maternally derived anti-NDV antibodies. In most vaccine groups, the titers of post-vaccination HI antibodies reached or surpassed the titer of maternal immunity at hatch, while the titers in the control groups decreased by 35%. The ability of ND vaccines to overcome maternal immunity is critical, as vaccine efficacy in birds is correlated with the level of maternally derived antibodies, which neutralize the vaccine and thereby reduce the effectiveness of vaccination [18,47,48,49]. Presence of maternal antibodies is known to interfere with the development of active immunity when ND vaccines are administered intramuscularly, subcutaneously, via the intranasal route, in drinking water, and by aerosol [9,12,18,50]. The protective efficiency of live ND vaccines after administration in chickens with maternal immunity through conjunctival and intranasal routes has been less impacted compared with the other routes, perhaps due to the development of local immunity induced by these vaccines [18,51]. The immunity induced by inactivated vaccines has also been less affected by the presence of maternal antibodies [52], specifically when high doses are used, but the limitation of these vaccines is their inability to induce mucosal immunity. It has also been demonstrated that the efficacy of recombinant NDV vector-based vaccines expressing avian influenza proteins was reduced when the vaccines were administered in birds with pre-existing anti-ND and anti-AIV antibodies [53,54,55]. In this study, as suggested by the increase in HI-antibody titers in the birds vaccinated with the experimental rNDV vaccines, a strong host humoral immune response was generated in spite of the presence of high maternal antibody levels.

The recombinant ND vaccines provided protection from early virulent NDV challenge. While the birds in all groups vaccinated with ZJ1*L, ZJ1*L-IL4R, or LS-IL4R attained 100% survival, the morbidity and mortality in the control groups were above 80% and 33%, respectively. The birds in the Hatch and BHI control groups were not fully protected when challenged with virulent ZJ1 NDV, displaying a high level of morbidity and mortality with lower after-challenge body weights compared with the rNDV vaccine groups. Although antibody titers above log_2_ 3 are generally considered protective [56,57], the high morbidity and mortality observed in the control birds (antibody titers log_2_ 4.5 at 13DPH) in the present study are suggestive that additional immune factors must play a role in the protection of birds from virulent challenge. It is possible that in absence of mucosal immunity, only maternally derived antibodies, even in titers above the protective cutoff, do not afford full protection from clinical disease and mortality. It has been reported that the *in ovo* route of vaccination presents the viral antigens to the mucosal surfaces of the respiratory tract and digestive tract favoring the development of mucosal immunity [58]. This, in addition to the high post-vaccination titers in the birds vaccinated with the recombinant experimental vaccines, could explain, in part, the 100% survival observed in these groups. Further studies to evaluate the level of mucosal immunity of *in ovo*-vaccinated birds is necessary to support this hypothesis. The rHVT-ND vaccine did not provide full protection, and relatively high percentages of morbidity (83.3%) and mortality (33.4) were observed. This is not surprising, as it has been reported that it takes up to four weeks before full immunity is reached with some rHVT-ND vaccines [31,59]. This delayed immunity has been attributed to the slower *in vivo* replication of the vector [19]. As anti-NDV maternal antibodies decline rapidly after hatch [1,60], this slower development of protective immunity induced by rHVT-ND vaccines may present a window of exposure, during which chickens are susceptible to vNDV infection. The clinical signs (predominantly neurological) that were observed in the Hatch, BHI, and rHVT-ND groups are indicative of suboptimal protection, likely afforded by the maternal antibodies.

The absence of significant changes in pre- and post-challenge HI antibody titers in the rNDV vaccinated groups is suggestive of low replication of the challenge virus and effective neutralizing immunity induced by the rNDV vaccines in these birds. The ZJ1*L, ZJ1*L-IL4R, and LS-IL4R vaccine group titers were all within one log difference compared with the pre-challenge titers. The decreased replication efficacy of the challenge virus in the vaccinated groups compared with the Hatch, BHI, and rHVT-ND control groups is additionally supported by the low amount viral RNA detected from the challenged birds inoculated with rNDV vaccines. This would strongly suggest that although humoral immunity has an important protective role, with these recombinant ND vaccines, the innate mucosal immunity, in association with the primed cell-mediated immunity, are likely responsible for neutralizing the challenge virus. To highlight this point, birds vaccinated with the rNDV experimental vaccines had significantly less viral RNA detected at 2 DPC and at 4 DPC. Indeed, at 2 DPC and 4 DPC, 25% to 50% of the rNDV experimentally vaccinated birds had no detectable viral RNA in samples. In contrast, significant post-challenge seroconversion was observed in the control Hatch, BHI, and rHVT-ND groups in which the HI antibody titers doubled as compared with pre-challenge, suggesting high replication of the challenge virus. Accordingly, shedding of high amounts of virus through the oral route at 2 DPC and through both routes at 4 DPC from the control birds was observed. In addition, the post-challenge body weights in all rNDV vaccinated groups were uniform and significantly higher compared with the Hatch, BHI, and rHVT-ND control groups. Although not significantly different, the titers of pre-challenge antibodies elicited by the LS-IL4R vaccines were one log lower than those elicited by the ZJ1*L-IL4R vaccines. It has been previously reported that anti NDV-antibodies more efficiently neutralize homologous viruses [16,17,61]. It is therefore possible that the differences in pre-challenge HI antibody titers are a result of more efficient inactivation of the LS-IL4R vaccine by maternal antibodies (LaSota was used to vaccinate the parents) compared with the ZJ1*L-IL4R vaccine.

The post-hatch survival in the groups vaccinated at 18 DOE with rNDV vaccines containing IL4R insert, although not significantly different, was dose-dependent. While some post-hatch mortality was observed in the groups vaccinated with the rNDV experimental vaccines, mortality decreased with decreasing vaccine dose. In the LS-IL4R 10^3.5^ group, the post-hatch survival was as high as the rHVT-ND control group. The post-hatch body weights in some of the vaccine groups were significantly lower at 8 and 13 DPH. However, the body weights in the ZJ1*L-IL4R 10^4.5^ and 10^5.5^ and LS-IL4R 10^3.5^ groups were only slightly and insignificantly lower compared with the control groups. These minimal post-hatch adverse effects could be mitigated by additionally decreasing the vaccine dose. The LS-IL4R vaccine provided 100% survival after early virulent challenge at the low 10^3.5^ dose, and evaluation of the protection efficacy of the recombinant ZJ1*L-IL4R vaccines using this and even lower doses is warranted. Moreover, the recombinant vaccines could be further attenuated by inserting an additional foreign gene [62].

The insertion of the antisense avian cytokine IL4 in the low virulent ZJ1*L and LS strains further attenuated these viruses, as was evident in the differences in post-hatch mortality and post-hatch body weights between the backbones and the recombinant experimental constructs. Insertion of foreign genes in the genome of NDV of low virulence has been shown to attenuate the virus with no adverse effects (e.g., the recombinant virus is not becoming more virulent or pathogenic) [62,63]. The expression of interferon gamma (IFNγ) by a virulent NDV attenuated the virus and decreased morbidity and mortality in SPF chickens [40]. Chicken IFN**γ**, which modulates macrophage activation and inhibits viral replication, has been shown to improve protection and enhance immune responses against different avian pathogens [39], including NDV [26]. A dangerous gain-of-function, such as increased virulence, has been observed with inserting IL4 in certain poxviruses [64]. In contrast, our study indicates a decrease in the virulence of the vector, which highlights the differences between viral vectors and probably the main pathways involved in the infection they cause. Although cytokine-expressing avian vaccines have been suggested to improve protective immunity [12], no vaccine product has yet become commercially available. Cytokines are components of a well-balanced system of immune responses with multiple feedback loops [19]. Modifying the balance in this system by up- or down-regulation of certain cytokines may direct the immune response in a desired direction [39], but additional studies to quantify the broad spectrum of interleukins, chemokines, and interferons are needed to properly evaluate the overall impact of vaccines containing cytokine inserts.

The results from the SPF control groups demonstrate that the observed differences in induced humoral immunity, shedding, and survival from early challenge with a virulent NDV do not appear to be influenced solely by the maternal antibody titers in these commercial egg-hatched birds. While there was significant morbidity, mortality, and seroconversion in the Hatch, BHI, and rHVT-ND groups, as well as marked clinical signs, the vaccinated challenged SPF groups had 100% survival, no morbidity, and minimal post-challenge seroconversion. These findings would appear to support the conclusion that the observed protection in the experimentally vaccinated groups is linked to the *in ovo* immunization (vaccination with rNDV vaccines) and not linked to the presence or titer of maternally derived antibodies. Furthermore, the observed active shedding of the virus in all the vaccinated birds demonstrates the successful uptake of the administered novel vaccines.

In summary, due to the global use of the *in ovo* method of vaccine administration and its multiple advantages to the poultry industry [19,20,21], the development of *in ovo* NDV-based vaccines holds great promise for the future control of this disease. Our results demonstrate the development of vaccines able to bypass maternal immunity elicit a strong immune response denoted by antibody titer as early as two weeks post-hatch (which coincides with the decay of maternal antibodies), provide full protection from clinical disease, and significantly decrease viral shedding after virulent challenge. This is the first report describing the *in ovo* use of live ND vaccines with high post-hatch survival rates and full protection from clinical signs after early virulent challenge. These novel vaccines are excellent candidates for further testing at lower doses and additional attenuation through reverse genetics and insertion of more genes. Due to their low cost and convenient production, storage, and transportation, such live rNDV vaccines may present an efficient alternative to current *in ovo* ND vaccines.

## Figures and Tables

**Table 1:** Mean weights (and standard deviations) of chickens at 1, 8, and 13 days post-hatch after inoculation of commercial eggs at 18 days of embryonation with experimental *in ovo* vaccines and at 14 days post-challenge with virulent Newcastle disease virus.

DPH or DPC	Mean Weights (Standard Deviations) of Chickens in Control and Vaccine Groups Presented in Grams											
	HATCH Control	BHI Control	rHVT-ND Control	LS 10^4.5^ Control	LS 10^5.5^ Control	ZJ1*L 10^4.5^	ZJ1*L 10^5.5^	ZJ1*L-IL4R 10^4.5^	ZJ1*L-IL4R 10^5.5^	LS-IL4R 10^3.5^	LS-IL4R 10^4.5^	LS-IL4R 10^5.5^
1 DPH	35.52 ^a,b^ (3.27)	39.67 ^a,b^ (2.31)	42.14 ^b,c^ (2.95)	38.50 ^a^ (3.00)	40.25 ^a,b^(3.178)	38.05 ^a^ (2.44)	40.21 ^a,b^ (1.90)	42.57 ^b,c^ (3.30)	40.71 ^a,b^ (2.64)	40.24 ^a,b^ (2.76)	39.05 ^a,b^ (2.66)	40.01 ^a,b^ (2.77)
8 DPH	76.81 ^a,b^ (9.95)	81.76 ^a,b^ (6.38)	81.95^a,b^ (7.46)	53.5 ^c^ (21.24)	NA	54.26 ^c^ (15.34)	58.06 ^c^ (6.74)	67.50 ^a,c^ (14.73)	69.85 ^a,c^ (13.24)	71.55 ^a,c^ (13.74)	63.33 ^c^ (14.19)	50.00 ^c^ (14.23)
13 DPH	123.80 ^a,b^(14.87)	123.50 ^a,b^ (9.67)	129.40^a,b^ (11.78)	95.75 ^c^ (27.18)	NA	99.59 ^c^ (19.21)	97.38^c^ (13.84)	113.40 ^a,c^ (13.88)	119.40 ^a,c^ (11.89)	116.70 ^a,c^ (23.30)	110.70^c^ (16.13)	88.93 ^c^ (24.88)
14 DPC	265.90 (65.85)	255.40 (67.3)	256.30 (86.05)	291.10 (56.29)	NA	299.90 (53.22)	289.50 (42.17)	296.90 (38.99)	328.30 (32.76)	305.90 (54.93)	313.80 (47.10)	282.30 (61.71)

The weights are presented in grams as mean values with standard deviations. Different lowercase superscript letters indicate statistically significant differences between groups (*p* value < 0.05). DPH = days post-hatch, DPC = days post-challenge, NA = not applicable, as all birds died before this measurement.

## Supplementary Materials

The following are available online at https://www.mdpi.com/article/10.3390/vaccines9101189/s1, Supplementary Figure S1: (A) Schematic representation of recombinant constructs and (B) schematic and (C) table representation of the study, Supplementary Figure S2: Survival of chickens post-hatch after inoculation of commercial eggs at 18 days of embryonation with experimental *in ovo* NDV vaccines and controls, Supplementary Figure S3: (A) Post-hatch survival, (B) mean post-hatch vaccine shedding titers, (C) pre- and post-challenge HI antibody titers, (D) post-challenge survival, and (E) mean post-challenge viral shedding titers after inoculation of SPF eggs at 18 days of embryonation with experimental *in ovo* vaccines and challenge with virulent Newcastle disease virus at 14 days post-hatch. SPF eggs were inoculated with ZJ1*L-IL4R 10^3.5^ and ZJ1*L 10^3.5^ EID_50_/per egg.
