# Supplementary material for: Novel Recombinant Newcastle Disease Virus-Based In Ovo Vaccines Bypass Maternal Immunity to Provide Full Protection from Early Virulent Challenge
Source: Vaccines (Basel). 2021 Oct 15;9(10):1189. doi: 10.3390/vaccines9101189 (PMC8538074; doi:10.3390/vaccines9101189)
Supplement: Supplementary file 1 [file vaccines-09-01189-s001.zip › Supplemental Figure S1.pdf]

Supplemental Figure S1A. Schematic representation of the final recombinant constructs: a) ZJ1*L-IL4R and b) LS-IL4R

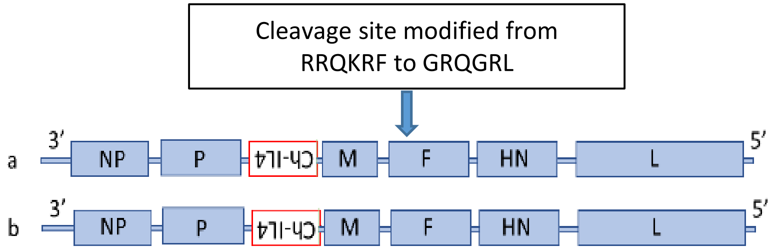

Supplemental Figure S1B. Schematic representation of the experiment

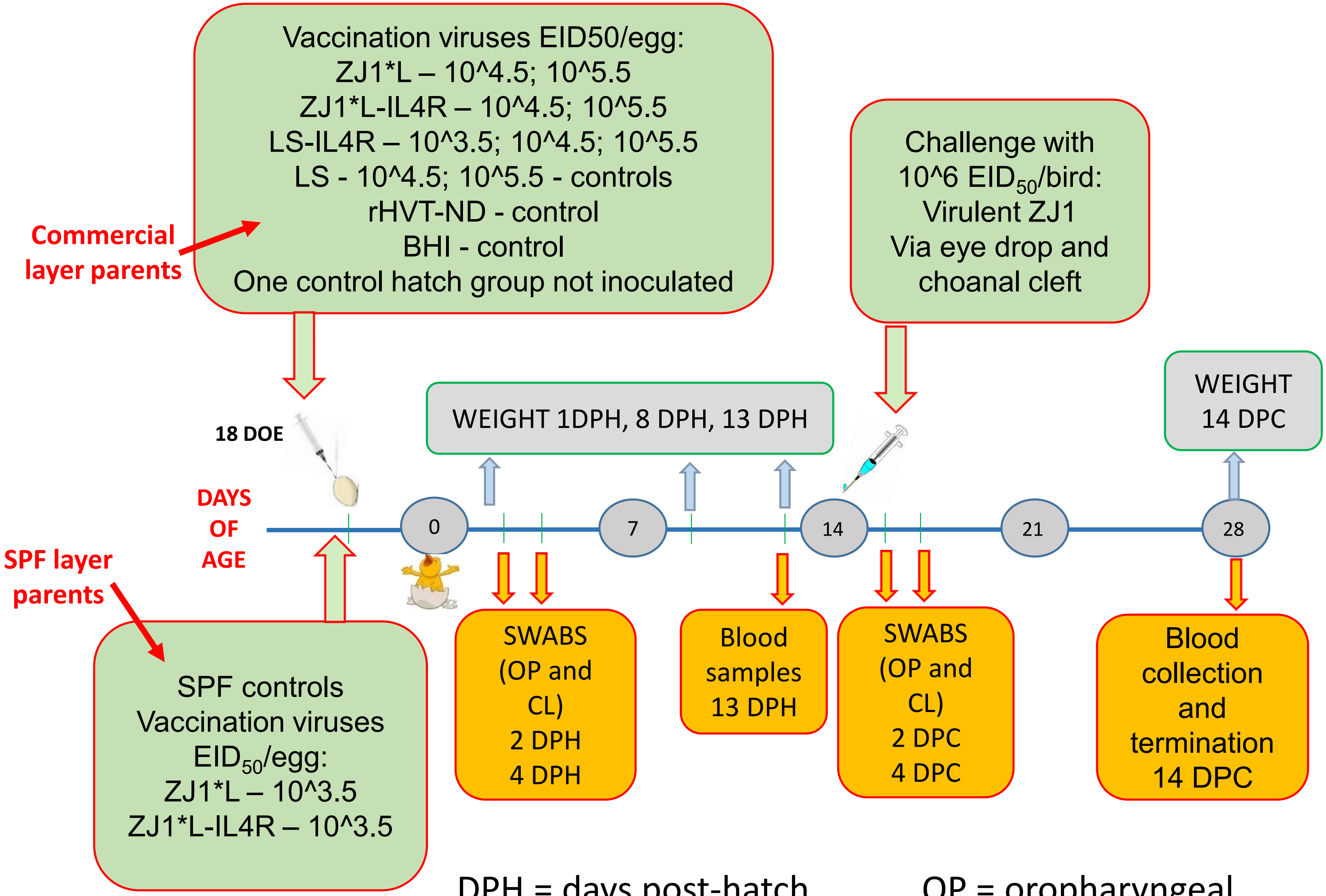

DPH = days post-hatch
DPC = days post-challenge
OP = oropharyngeal
CL = cloacal

Supplemental Figure S1C. Table representation of the experiment with groups

<table>
<tr><th>Group</th><th>Vaccine/inoculum
0.1mL
Oculo-nasal</th><th>Dose
EID_{50}/0.1mL</th><th>Number of hatched eggs</th><th>Bleeding for maternal antibody levels</th><th>Weight measuring</th><th>Swab collection (OP and CL)</th><th>Blood collection</th><th>Challenge with virulent NDV 10^6 EID_{50}/bird</th><th>Swab collecti on (OP and CL)</th><th>Blood collection</th></tr>
<tr><td>1</td><td>None – hatch control</td><td>-</td><td>26</td><td>5 birds at hatch</td><td rowspan="14">1,8, 13 DPH</td><td rowspan="14">2 and 4 DPH</td><td rowspan="13">13 DPH</td><td rowspan="13">14 DPH</td><td rowspan="13">2 and 4 DPC</td><td rowspan="13">14 DPC</td></tr>
<tr><td>2</td><td>BHI control</td><td>-</td><td>26</td><td>5 birds at hatch</td></tr>
<tr><td>3</td><td>rHVT-ND control</td><td>Manufacturer recommendations</td><td>21</td><td rowspan="12">NONE</td></tr>
<tr><td>4</td><td>ZJ1*L</td><td>10^4.5</td><td>21</td></tr>
<tr><td>5</td><td>ZJ1*L</td><td>10^5.5</td><td>21</td></tr>
<tr><td>6</td><td>ZJ1*L-IL4R</td><td>10^4.5</td><td>21</td></tr>
<tr><td>7</td><td>ZJ1*L-IL4R</td><td>10^5.5</td><td>21</td></tr>
<tr><td>8</td><td>LS-IL4R</td><td>10^3.5</td><td>21</td></tr>
<tr><td>9</td><td>LS-IL4R</td><td>10^4.5</td><td>21</td></tr>
<tr><td>10</td><td>LS-IL4R</td><td>10^5.5</td><td>21</td></tr>
<tr><td>11</td><td>ZJ1*L – SPF</td><td>10^3.5</td><td>20</td></tr>
<tr><td>12</td><td>ZJ1*L-IL4R – SPF</td><td>10^3.5</td><td>21</td></tr>
<tr><td>13</td><td>LS control</td><td>10^4.5</td><td>21</td></tr>
<tr><td>14</td><td>LS control</td><td>10^5.5</td><td>19</td><td>DEAD</td><td>NA</td><td>NA</td><td>NA</td></tr>
</table>

DPH = days post-hatch
DPC = days post-challenge
NA = not applicable
OP = oropharyngeal
CL = cloacal
DEAD = all birds died by this day
