# Supplementary material for: Novel Recombinant Newcastle Disease Virus-Based In Ovo Vaccines Bypass Maternal Immunity to Provide Full Protection from Early Virulent Challenge
Source: Vaccines (Basel). 2021 Oct 15;9(10):1189. doi: 10.3390/vaccines9101189 (PMC8538074; doi:10.3390/vaccines9101189)

Supplemental Figure S2. Survival of chickens post-hatch after inoculation of commercial eggs at 18 days of embryonation with experimental in ovo NDV vaccines and controls.

Hy-Line Rockside W-36 eggs were inoculated with recombinant viruses and LaSota. Three control groups were inoculated with BHI or rHVT-ND vaccine, or not inoculated (Hatch control group). Birds were monitored for 13 days post-hatch. Different superscript letters indicate statistically significant differences between groups (p value <0.05). Group abbreviations: HATCH – eggs not inoculated; BHI – eggs inoculated with BHI; rHVT-ND – eggs inoculated with recombinant HVT vaccine; ZJ1*L – eggs inoculated with the recombinant ZJ1*L virus whose cleavage site was modified to be of low virulence; ZJ1*L-IL4R – eggs inoculated with the recombinant low virulent ZJ1*L with the interleukin-4 gene inserted in reverse orientation; LS – eggs inoculated with the LaSota strain; LS-IL4R – eggs inoculated with the recombinant LaSota virus with the interleukin-4 gene inserted in reverse orientation. The virus dose that the eggs in each group received is represented as a number after the group name in EID50/per egg.

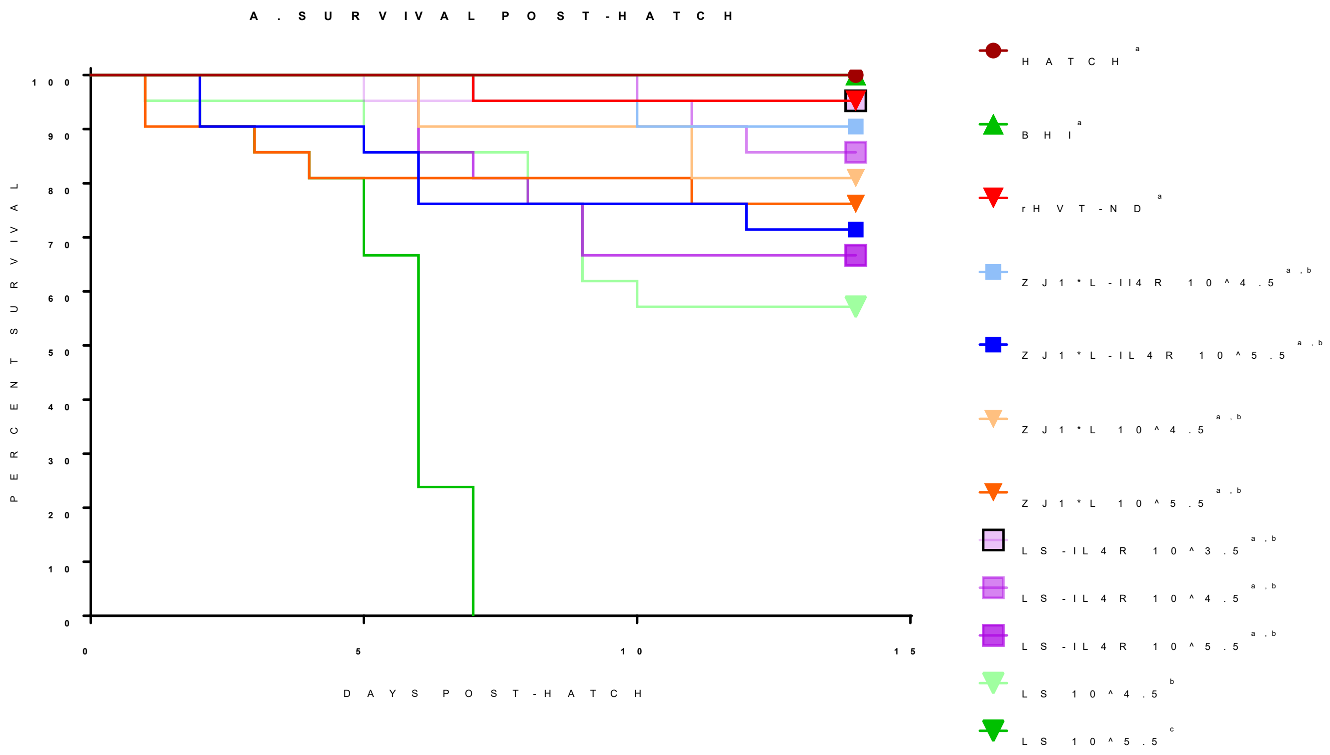

Supplement: Supplementary file 1 [file vaccines-09-01189-s001.zip › Supplemental Figure S2.pdf]
