# Supplementary material for: Novel Recombinant Newcastle Disease Virus-Based In Ovo Vaccines Bypass Maternal Immunity to Provide Full Protection from Early Virulent Challenge
Source: Vaccines (Basel). 2021 Oct 15;9(10):1189. doi: 10.3390/vaccines9101189 (PMC8538074; doi:10.3390/vaccines9101189)

Supplemental Figure S3A. Survival of chickens post-hatch after inoculation of SPF eggs at 18 days of embryonation with experimental *in ovo* vaccines.

The eggs were inoculated with ZJ1*L-IL4R $10^{3.5}$ and ZJ1*L $10^{3.5}$ $EID_{50}$/per egg. Control groups were inoculated with BHI or rHVT-ND vaccine, or left not inoculated (hatch control).

The data for the HATCH, BHI and rHVT groups are the same as Figure 1 and are presented here for comparison purposes.

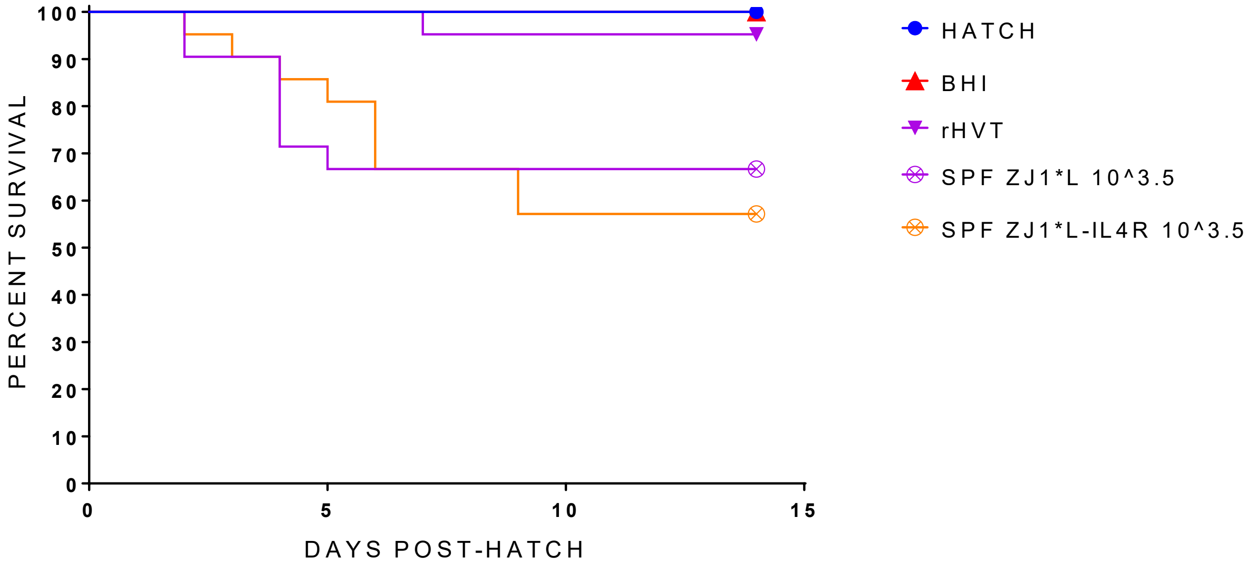

Supplemental Figure S3B. Mean post-hatch vaccine shedding from chickens hatched from SPF eggs inoculated at 18 days of embryonation with experimental *in ovo* vaccines. Shedding is presented separately at 2 and 4 days post-hatch. Each data point represents NDV titers detected in oropharyngeal (OP) and cloacal (CL) swabs at different days post-hatch (DPH). Bars represent standard deviations of the mean. All swabs from which virus was not detected were given a numeric value of 1 log below the limit of detection for each of the respective viruses. The limit of detection of rRT-PCR is presented as a horizontal dotted line (the lowest limit is used).

The eggs were inoculated with ZJ1*L-IL4R $10^{3.5}$ and ZJ1*L $10^{3.5}$ $EID_{50}$/per egg. Control groups were inoculated with BHI or rHVT-ND vaccine, or left not inoculated (hatch control). The data for the HATCH and BHI groups are the same as Figure 2 and are presented here for comparison purposes.

## VACCINE SHEDDING AT DAY 2 POST-HATCH

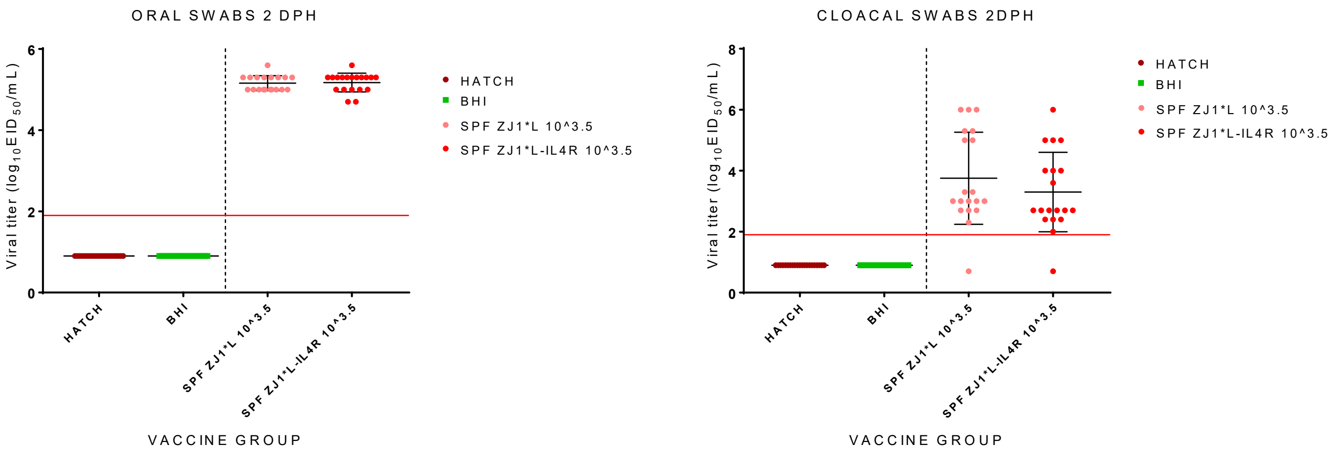


## VACCINE SHEDDING AT DAY 4 POST-HATCH

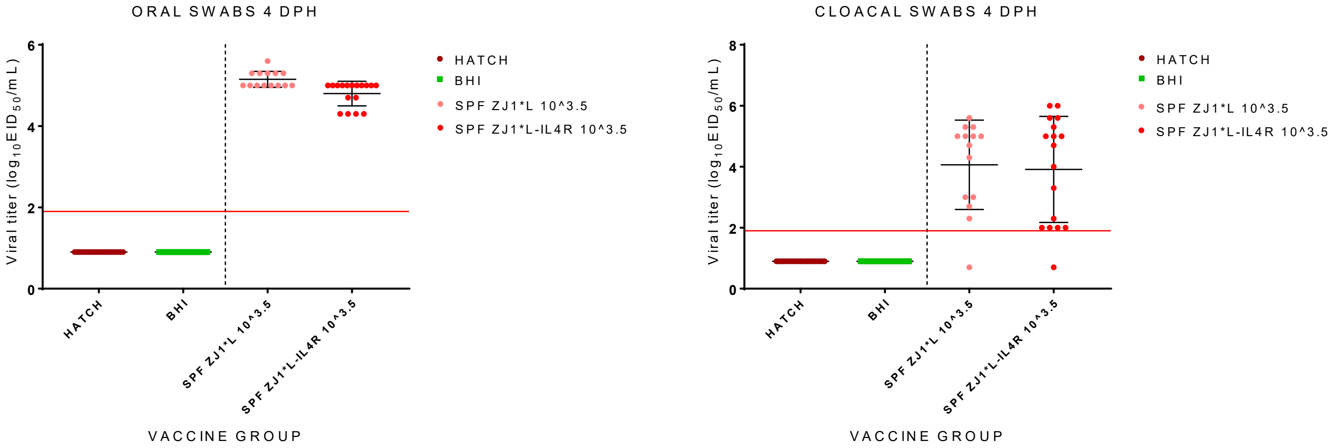

Supplemental Figure S3C. Pre- and post-challenge HI antibody titers (and standard deviation) from chickens hatched from SPF eggs inoculated at 18 days of embryonation with experimental *in ovo* vaccines and early challenge with virulent Newcastle disease virus at 14 days post-hatch.

The diagonal stripe filled bars represent the pre-challenge titers at 13 days post-hatch. The solid filled bars represent the post-challenge titers at 14 days post-challenge. Statistically significant differences between the different groups are presented with lowercase letters above each group (*p* value <0.05). The data for the HATCH, BHI and rHVT groups are the same as Figure 3 and are presented here for comparison purposes.

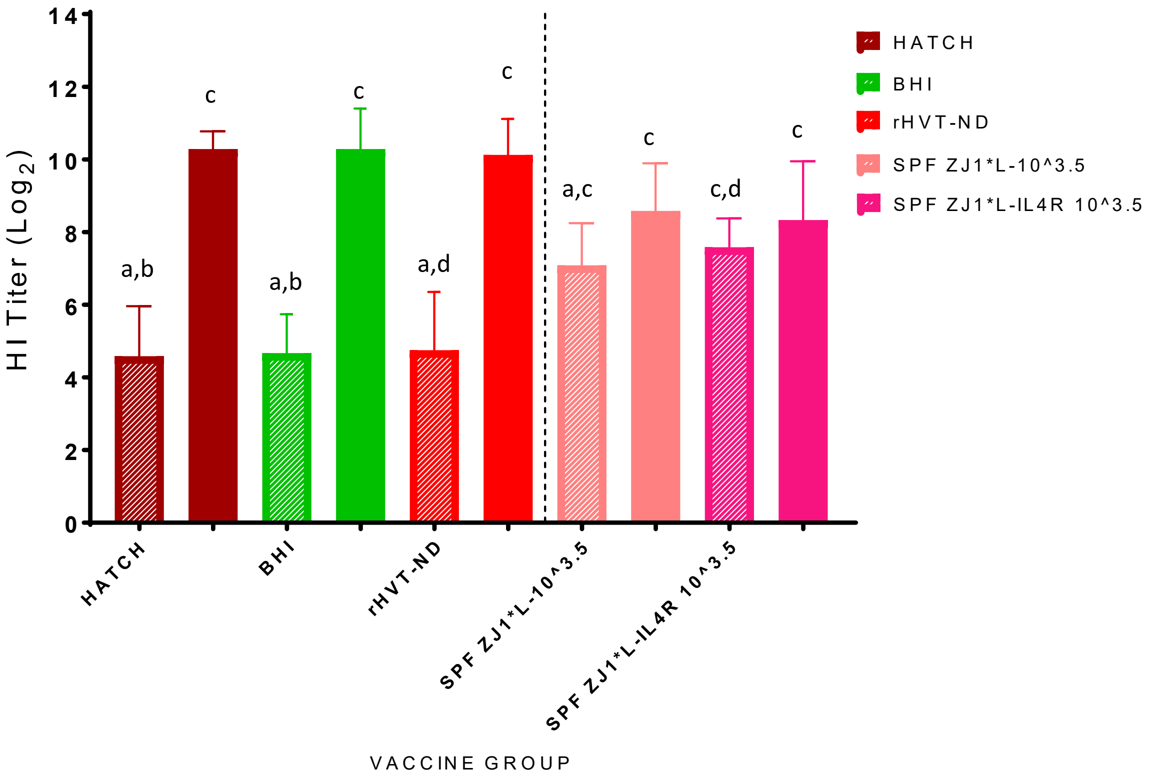

Supplemental Figure S3D. Survival of chickens after inoculation of SPF eggs at 18 days of embryonation with experimental *in ovo* vaccines and early challenge with virulent Newcastle disease virus at 14 days post-hatch.

Birds were monitored for 14 days post-challenge. Different superscript letters indicate statistically significant differences between groups ($p$ value <0.05). The data for the HATCH, BHI and rHVT groups are the same as Figure 4 and are presented here for comparison purposes.

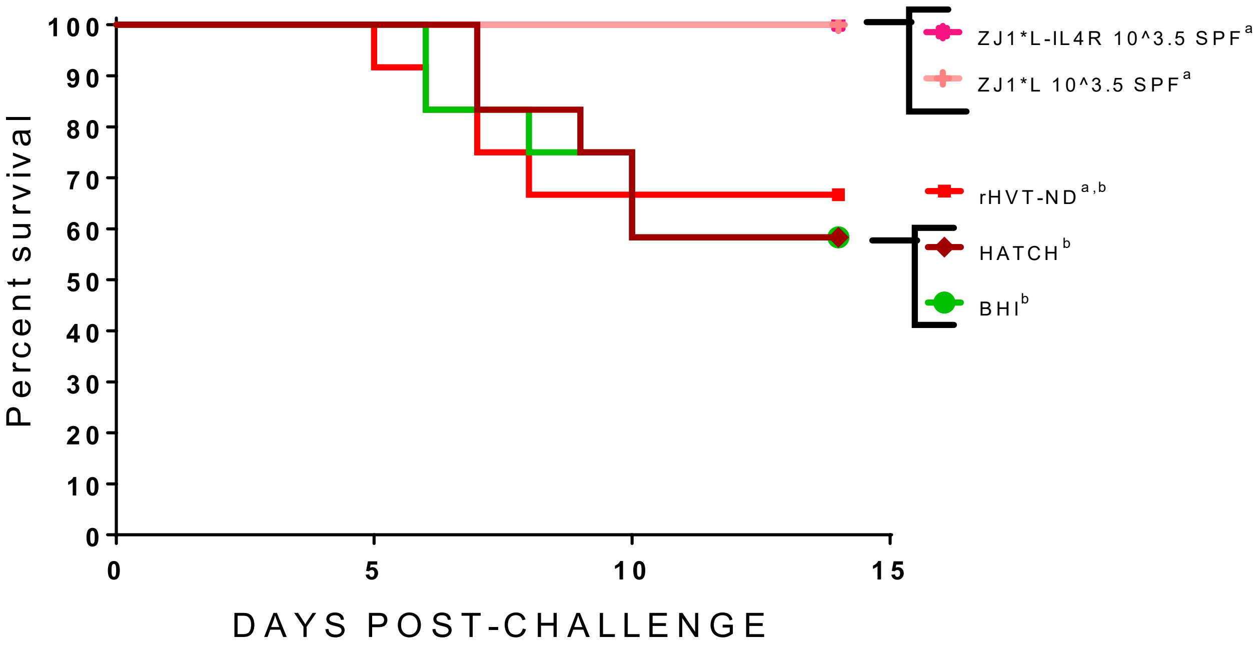

Supplemental Figure S3E. Mean post-challenge viral shedding from chickens after inoculation of SPF eggs at 18 days of embryonation with experimental *in ovo* vaccines and early challenge with virulent Newcastle disease virus at 14 days post-hatch.

Each data point represents NDV titers detected in oropharyngeal (OP) and cloacal (CL) swabs at different days post-challenge (DPC). Bars represent standard deviations of the mean. All swabs from which virus was not detected were given a numeric value of 1 log below the limit of detection. The limit of detection of rRT-PCR is presented as a horizontal dotted line. Statistically significant differences between the different groups are presented with lowercase letters above each group ($p$ value <0.05). The data for the HATCH, BHI and rHVT groups are the same as Figure 5 and are presented here for comparison purposes.

## VIRAL SHEDDING AT DAY 2 POST-CHALLENGE

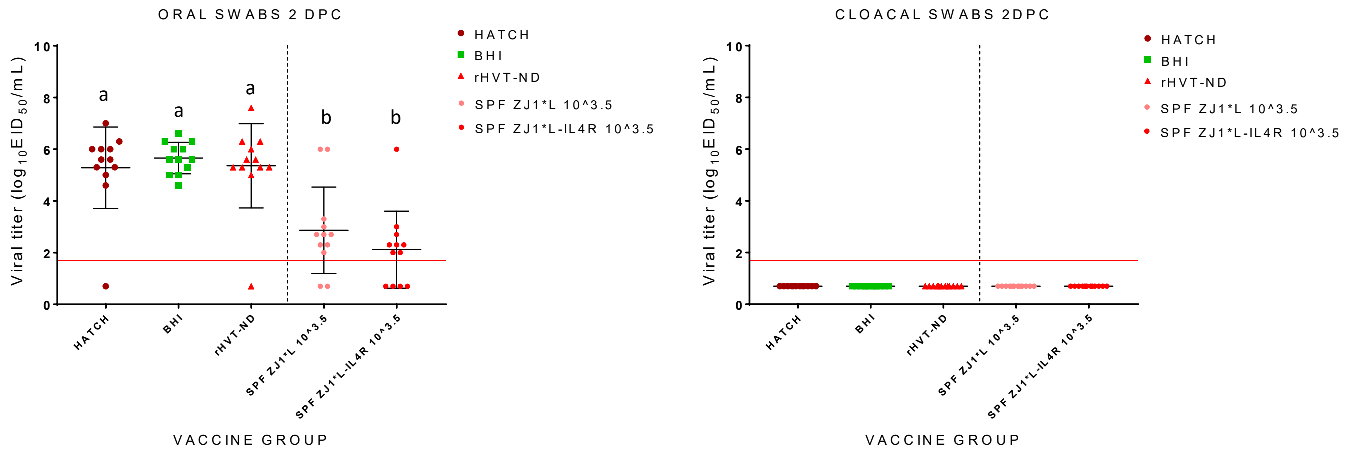


## VIRAL SHEDDING AT DAY 4 POST-CHALLENGE

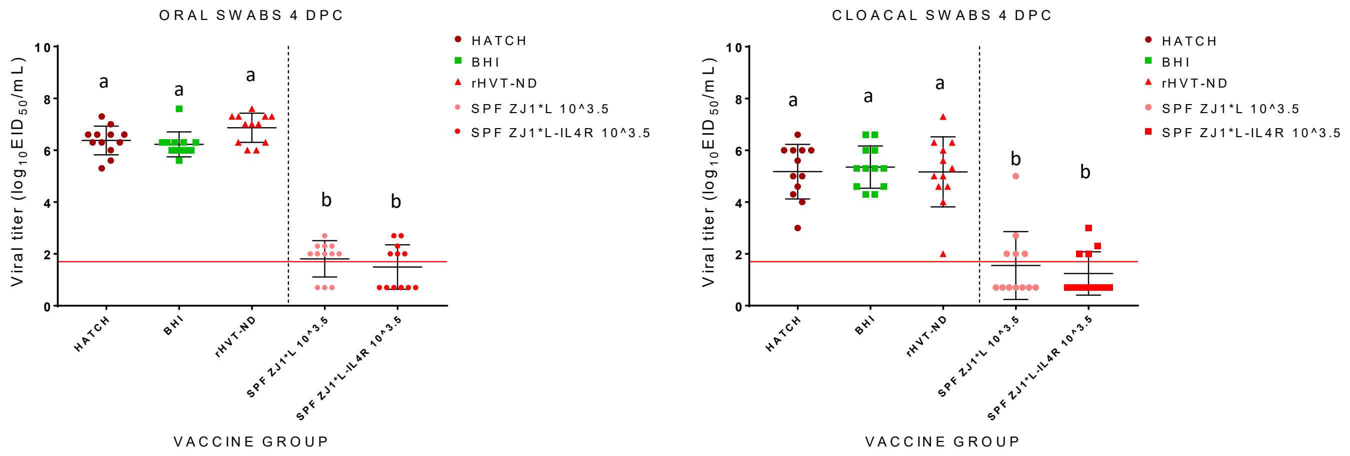

Supplement: Supplementary file 1 [file vaccines-09-01189-s001.zip › Supplemental Figure S3.pdf]
